# Supplementary figures and images for: Deep Neural Networks Rival the Representation of Primate IT Cortex for Core Visual Object Recognition
Source: PLoS Comput Biol. 2014 Dec 18;10(12):e1003963. doi: 10.1371/journal.pcbi.1003963 (PMC4270441; doi:10.1371/journal.pcbi.1003963)

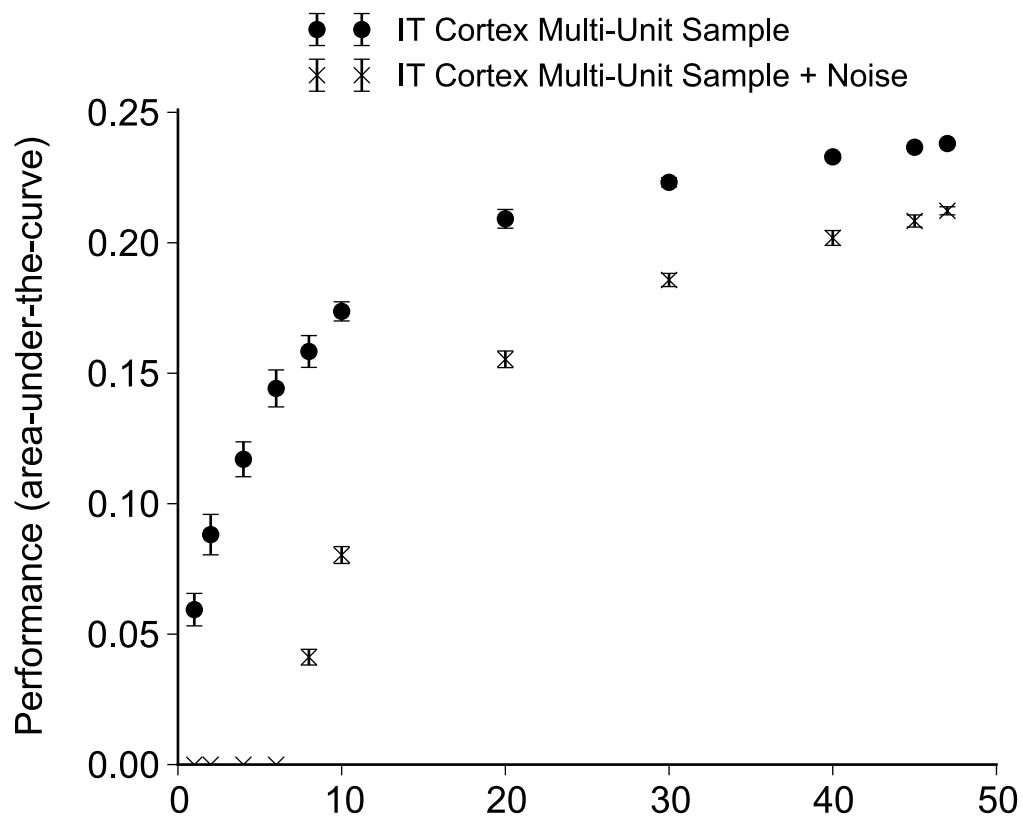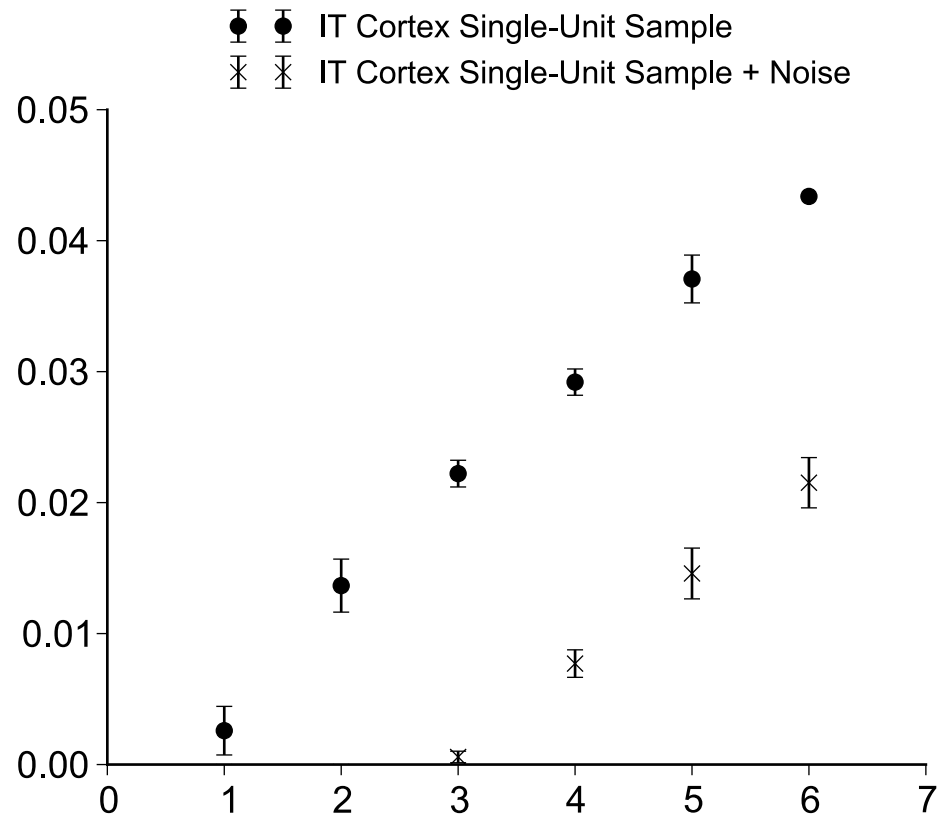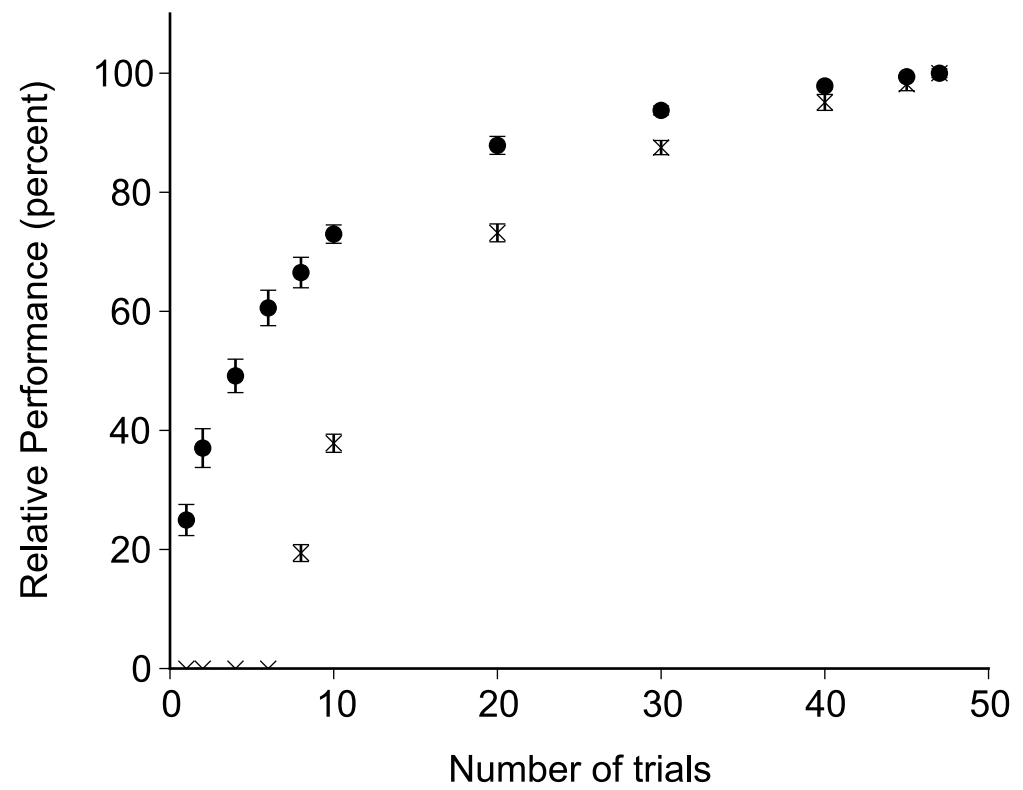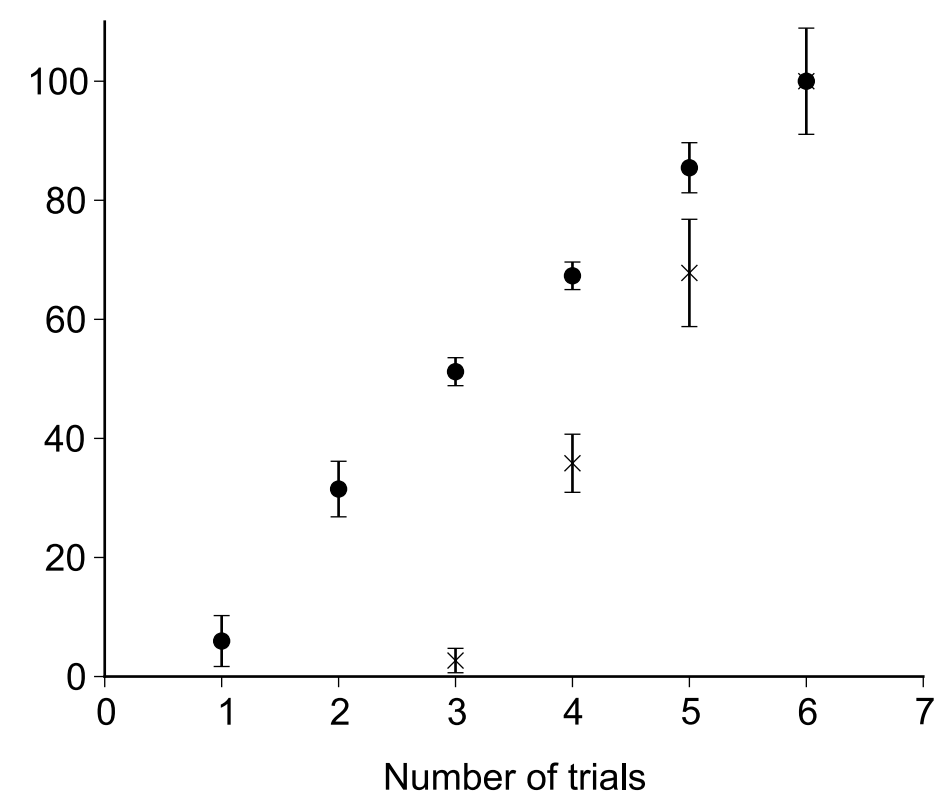

Supplement: S1 Fig — Effects on kernel analysis performance of empirical noise vs. induced noise model. In the top left panel we show the performance measurements, as measured by kernel analysis area-under-the-curve, of the IT cortex multi-unit sample and of the IT cortex multi-unit sample with trial dependent added noise as we vary the number of experimental trials (repetitions per image) or the trials in the noise model ( in Eq. 10). In all plots error bars indicate standard deviations of the measure over 10 repetitions of the analysis. Results are replotted and divided by the maximum performance (Relative Performance) in the lower left panel. The same analysis is performed for the IT cortex single-unit sample in the right panels. These results indicate that the noise model reduces our performance measurement over the empirically observed noise and is therefore a conservative model for inducing noise in model representations. In other words, these results indicate that the model representations with neural matched noise are likely overly penalized. (PDF) [file pcbi.1003963.s001.pdf]

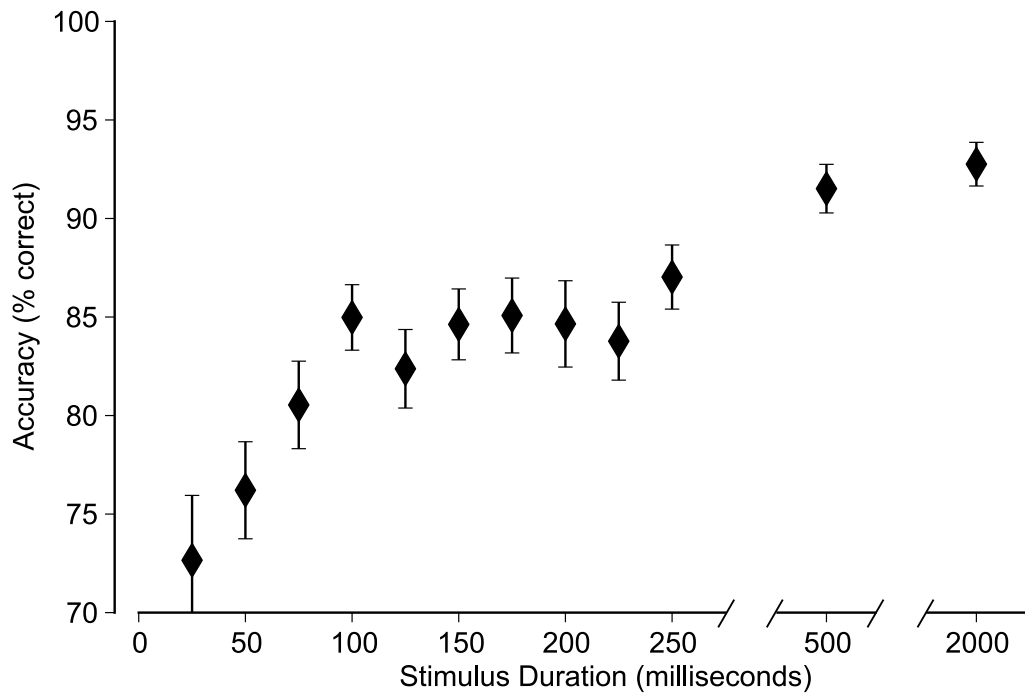

Supplement: S2 Fig — Human performance on the visual recognition task as a function of presentation time. We plot the mean block-accuracy for different stimulates presentation durations from responses measured using Amazon Mechanical Turk. The mean accuracy is plotted as diamond markers and the error bars indicate the 95% confidence interval of the standard error of the mean over block-accuracies. Chance performance is ∼14% for this task. The accuracy quickly increases such that at 100 ms stimulus duration it is within 92% of the performance at 2 seconds. This indicates that on this task, human subjects are able to perform relatively highly even during brief presentations of 100 ms. We refer to this ability as “core visual object recognition” [6] and we seek to measure the neural representational performance that subserves this ability. (PDF) [file pcbi.1003963.s002.pdf]

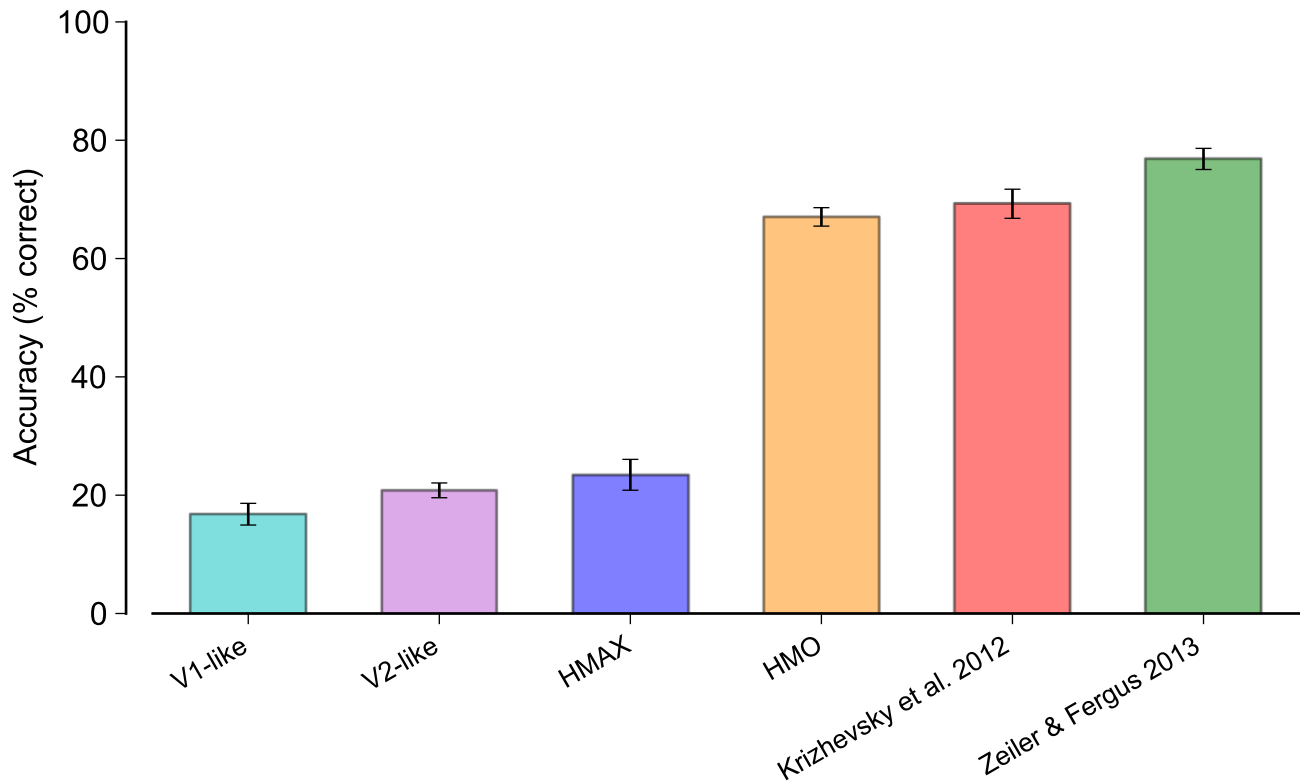

Supplement: S3 Fig — Linear-SVM performance of model representations without sample or noise correction. Testing set classification accuracy averaged over 10 randomly-sampled test sets is plotted and error bars indicate standard deviation over the 10 random samples. Chance performance is ∼14.3%. Unlike in Fig. 5, the model representations in this figure has not been modified to correct for sampling or noise. (PDF) [file pcbi.1003963.s003.pdf]

A

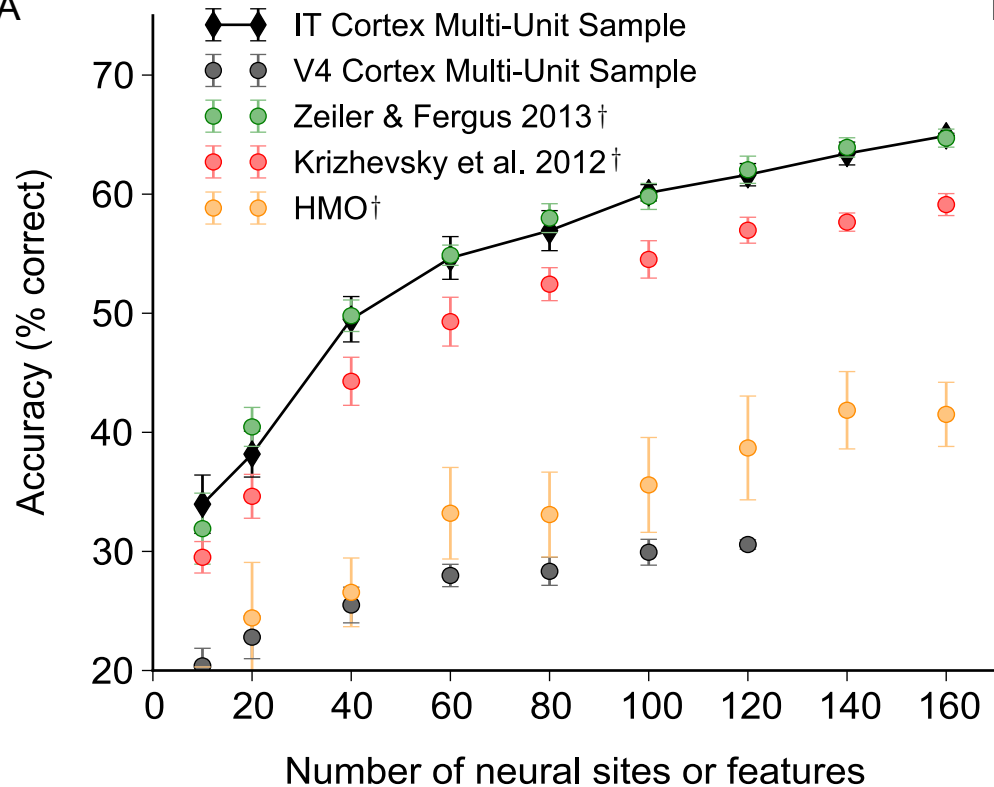

B

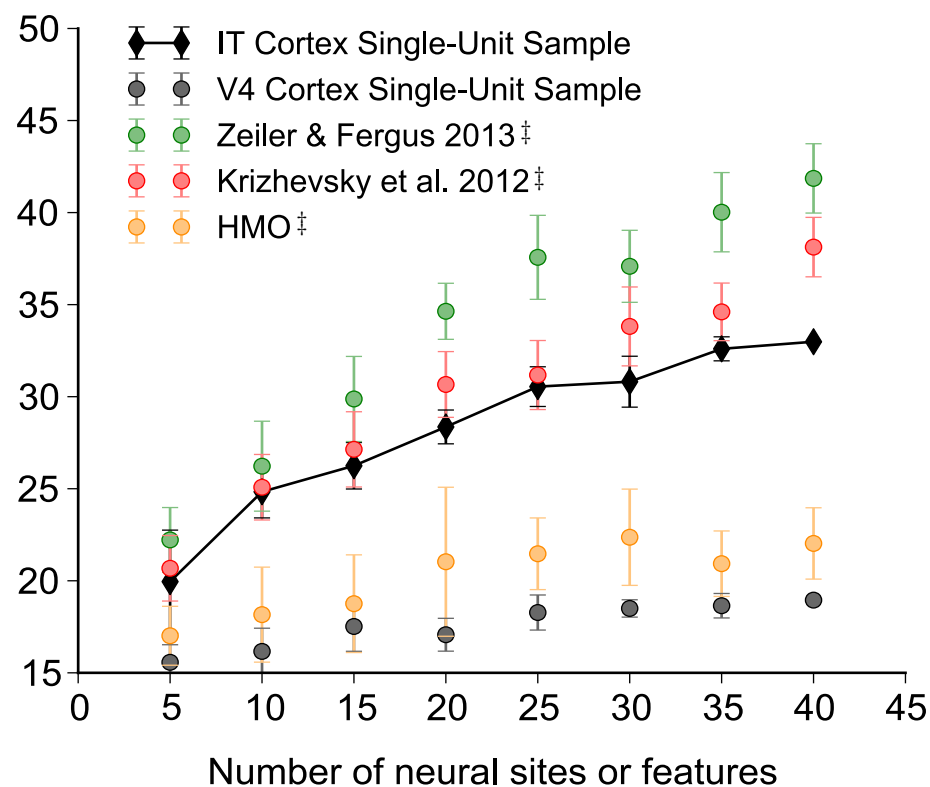

Supplement: S4 Fig — Effect of sampling the neural and noise-corrected model representations for the linear-SVM analysis. We measure the mean testing-set linear-SVM generalization performance as we change the number of neural sites (for neural representations), or the number of features (for model representations). Measured samples are indicated by filled symbols and measured standard deviations indicated by error bars. Multi-unit analysis is shown in panel A and single-unit analysis in B. The model representations are noise corrected by adding noise that is matched to the IT multi-unit measurements (A, as indicated by the symbol) or single-unit measurements (B, as indicated by the symbol). This analysis reveals a similar relationship to that found using the kernel analysis methodology (compare to Fig. 5). (PDF) [file pcbi.1003963.s004.pdf]
