# Supplementary material for: Deep Neural Networks Rival the Representation of Primate IT Cortex for Core Visual Object Recognition
Source: PLoS Comput Biol. 2014 Dec 18;10(12):e1003963. doi: 10.1371/journal.pcbi.1003963 (PMC4270441; doi:10.1371/journal.pcbi.1003963)
Supplement: S5 Fig — Comparison of IT multi-unit and single-unit representations. In the left panel we plot the kernel analysis AUC as a function of the number of single- or multi-unit sites. We plot results for two single-unit samples. “IT Cortex Single-Unit All-SNR Sample” uses all 160 isolated single-units and “IT Cortex Single-Unit High-SNR Sample” uses the 40 most consistent (least noisy) single-units. In the right panel we show the same data, but correct the number of multi-units to reflect an estimate of the number of single-units contributing to each multi-unit recording, thus plotting against the number of estimated neurons. Unlike previous figures, these estimates have a fixed number of trials (6) for both single- and multi-unit samples. Surprisingly, multi-unit recordings surpass single-unit recordings in performance (left) and are five times better per unit in performance to the all SNR single-unit sample (right). (PDF) [file pcbi.1003963.s005.pdf]

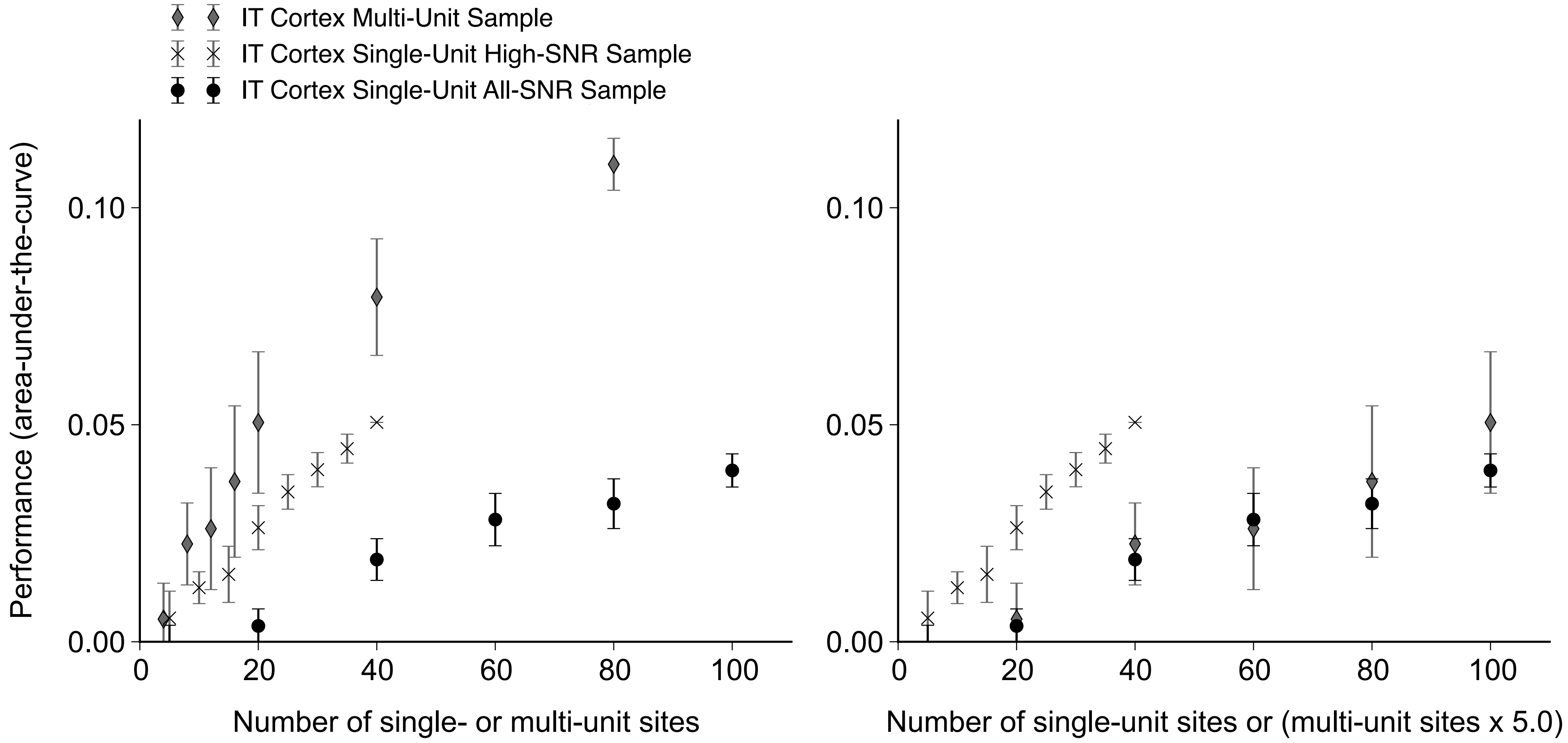

IT Cortex Multi-Unit Sample
IT Cortex Single-Unit High-SNR Sample
IT Cortex Single-Unit All-SNR Sample
Performance (area-under-the-curve)
0.00
0.05
0.10
0
20
40
60
80
100
Number of single- or multi-unit sites
Number of single-unit sites or (multi-unit sites x 5.0)
