# Supplementary material for: Deep Neural Networks Rival the Representation of Primate IT Cortex for Core Visual Object Recognition
Source: PLoS Comput Biol. 2014 Dec 18;10(12):e1003963. doi: 10.1371/journal.pcbi.1003963 (PMC4270441; doi:10.1371/journal.pcbi.1003963)
Supplement: S1 Text — Supporting Information for Methods and Discussion. Validation of the experimental noise matched model; Human performance on our task as a function of presentation time; Linear-SVM methodology; Comparing IT multi-unit and single-unit representations; Processing time and energy consumption of computational models. (PDF) [file pcbi.1003963.s006.pdf]

Deep Neural Networks Rival the Representation of Primate IT Cortex for Core Visual Object Recognition

Charles F. Cadieu[1,*], Ha Hong[1,2], Daniel L. K. Yamins[1], Nicolas Pinto[1], Diego Ardila[1], Ethan A. Solomon[1], Najib J. Majaj[1], James J. DiCarlo[1]
1 Department of Brain and Cognitive Sciences and McGovern Institute for Brain Research, Massachusetts Institute of Technology, Cambridge, MA 02139
2 Harvard–MIT Division of Health Sciences and Technology, Institute for Medical Engineering and Science, Massachusetts Institute of Technology, Cambridge, MA 02139
∗ E-mail: Corresponding cadieu@mit.edu

Supporting Information

Validation of the experimental noise matched model

To determine the effect of experimental and neural variability we measured the result of reducing experimental trials and varying the trials in Eq. 10 of the noise model. These results are shown for both multi-unit and single-unit IT cortex samples in Figure S1. The measurements of IT cortex multi-unit sample and single-unit sample show the effect of noise as empirically observed from increasing the number of trials (47 total trials for the multi-unit sample and 6 trials for the single-unit sample). We measured the effect of our noise model by starting with the IT cortex sample (s_{lj} in Eq. 10) at the maximum recorded trials, and added noise according to Eq. 10 while varying the number of trials in the model (T in Eq. 10). Not surprisingly this produces a reduction in measured performance. Importantly, the rate of performance decrease is larger in magnitude for the noise model than the observed empirical effect of reducing the number of trials. This can be seen by examining the relative performance in Figure S1. Therefore, the noise model is a conservative model for inducing noise in model representations because it overly penalizes a representation as we reduce the number of trials or, equivalently, increase the amount of noise.

Human performance on our task as a function of presentation time

In this section we present measurements of human performance on our task that contribute to our justification for measuring the neural representation at 100 ms presentation times. A number of criteria influence our choice of presentation time for the neural measurements, which for the following reasons we have chosen to be 100 milliseconds (ms). First, we seek to address a sub-problem in general visual behavior: core visual object recognition [1], or visual information processing within one saccade without contextual influence. The typical time between saccades is approximately 200-250 ms under natural viewing conditions [2] and we therefore require a presentation time equal to or less than 200 ms. Second, to improve the throughput of our neural recordings we desire to minimize the presentation time. However, in opposition of minimizing the presentation time for throughput, our final criteria is to choose a presentation time for which the primate visual system is still performant. If we were to reduce the presentation time toward 0 ms we expect the human behavioral performance on this task to approach chance. Therefore we do not want to reduce the presentation time so much that the primate system fails to perform the relevant visual behavior. To address this issue, we conducted a psychophysical experiment to evaluate the effect of presentation time on performance for our object recognition task, which we describe next. In summary, given these three criteria we seek a presentation time that is within the time of a typical saccade, and is long enough that the primate visual system is still performant at that presentation time.

To justify our selection of the presentation time of 100 ms, we measured human subjects using Amazon Mechanical Turk on our visual category object recognition task for different presentation times (Figure S2). The results of this experiment indicate that human behavioral performance reaches a mean accuracy

of 92.8% for 2 second presentation times. While it might be a priori expected that humans would reach 100% accuracy for 2 second presentation times, the difficultly of this task results in reduced performance. From manual examination of typical errors, image instances that have non-prototypical poses of objects (e.g. the underside of a car) or that have the object instance occluded by the image boundary lead to errors. Regardless of this ceiling effect, we find that human performance is quite robust to reduced presentation times. We observe that mean performance at 50 ms presentations reaches 82% of the performance at 2000 ms presentations. We expect that if we continue to decrease the presentation time the human performance will necessarily approach chance performance of ∼14%. However, instead of a linear decrease in performance as we reduce the presentation time, we see a saturation effect in performance with the majority of the performance obtained for presentations of 50 ms. For our chosen presentation time of 100 ms for the neural recordings we observe that mean human performance is 92% of the 2000 ms presentation time performance. Furthermore, the performance at 100 ms is close to the performance at 200 ms (the mean time of a saccade). Therefore, justifying our neural recording presentation time, 100 ms is within typical fixational eye-movements, 100 ms allows us to nearly double our throughput for data collection over 200 ms, and the primate visual system is able to achieve high performance on this task at 100 ms, making it a relevant behavioral regime for study.

The details of the human behavioral measurements are as follows. We recruited subjects through Amazon Mechanical Turk, an online platform where subjects can complete short experiments for small payments. To increase data reliability, all subjects had a prior task approval rating of 90%; they were approved for payment on at least nine out of ten tasks they had ever done previously on Mechanical Turk. Each subject was presented with a target image at the center of their screen, followed by a 500 ms delay. Subjects were then asked to click one of 7 response images (backgroundless canonical views of category exemplars) that matched the category of the target image. After giving a response, subjects were shown a central fixation point for 500 ms before the next target image appeared. Participating subjects were required to complete at least one block of trials, but were allowed to complete an unlimited number of additional blocks. Each block of trials was presented with the same stimulus duration, which ranged between 25 ms and 2000 ms, and the number of trials within each block was chosen to keep the amount of time required for each block equal between stimulus duration conditions. This had the effect of keeping the effective wage rate equal between conditions, which was approximately $10/hour. We additionally manually and algorithmically screened the data for cheating (for example, providing the same response to every target image, providing strings of identical responses, or failing to produce high entropy in the response distribution). After screening the dataset consisted of 96,717 total responses. For each block of trials we took the accuracy over the trials and excluded the first 5 responses. The mean of the block accuracies for each presentation time are shown in Figure S2 and the error bars indicate the 95% confidence interval of the standard error of the mean over block-accuracies. As a further check for the stimulus duration lengths presented by the subject's computer system, we measured the stimulus durations presented by a number of computer setups. We used a photodiode attached to an oscilloscope and measured the response of the monitor for 50 and 100 millisecond presentations, using 4 different computers and 3 different browsers. We found a standard deviation of measured presentation time of 4.95 ms for the 50ms presentation time and 10.3 ms for the 100ms presentation time. While this variation could be reduced by more controlled software and hardware environments, we concluded the amount of variation was acceptable for our measurements given the benefit of using Amazon Mechanical Turk to achieve many more samples at lower cost than in laboratory experiments.

Linear-SVM methodology

For the linear support vector machine methodology (linear-SVM) we seek to measure the generalization performance of a linear classifier estimated on one subset of the data, the training set, and measured on another, the testing set. By choosing a linear classifier the decision boundary is a simple linear function. By imposing this simplicity on the classifier, effective representations will be those that allow this simple

classifier to achieve high generalization performance, and performance is not confounded by the additional functional complexity introduced by a more complex decision function. This procedure therefore requires the representation, rather than the decision function, to "solve" the problem.

In detail we train a linear support vector machine on 80% of the images (1960 images total, 1568 training, 392 testing) using the scikit-learn (`http://scikit-learn.org`) wrapper to LIBLINEAR (`http://www.csie.ntu.edu.tw/~cjlin/liblinear`). We used a squared hinge loss, a one versus rest procedure, a squared penalty on parameters, and selected the regularization parameter (C) by 3-fold cross-validation on the training set, and then re-estimating the linear-SVM using the optimal value for C on the entire training set. We then used the trained linear SVM to predict the categories of the testing images and report the mean classification accuracy over the testing set. To measure statistical variation due to subsampling of image variation parameters we compute the testing set accuracy ten times, each time sampling 80% of the images for training and the remaining for testing. The ten samples are fixed for all representations and within each randomization we equalize the number of images from each category.

In Figure S3 we present the performance of the linear-SVM methodology on the model representations without correction for noise or trials. In Figure S4 we present the linear-SVM analysis as a function of sampling. Figure S4 is analogous to Figure 4, but uses the linear-SVM methodology instead of the kernel analysis methodology.

Comparing IT multi-unit and single-unit representations

Here we directly compare the representational performance between the IT multi-unit sample and the IT single-unit sample. Our previous analyses did not correct for the wide difference in trials between our multi- and single-unit samples, which have 47 and 6 trials, respectively. In Figure S5 we show the results of the kernel analysis area-under-the-curve measurements as a function of the number of single- or multi-unit sites and fixing each representation to 6 trials (responses are averaged over 6 randomly sampled trials). For the single-unit sample we show the analysis using all 160 isolated single-units ("IT Cortex Single-Unit All-SNR Sample") and the 40 most consistent (highest SNR) single-units ("IT Cortex Single-Unit High-SNR Sample"). In the left panel of Figure S5 we directly compare the number of multi-units to the number of single-units and show that the multi-unit representation outperforms the single-unit representation. We can also introduce another correction between the multi-unit and single-units by attempting to compare directly the number of neurons that go into each measurement. By using an independent dataset [3] collected in our lab using single-electrode electrophysiology and comparing to our multi-electrode setup, we determine based on spike counts that each multi-unit in our sample is approximately 4-5 single-units. Therefore, in the right panel in Figure S5 we plot the results by multiplying the number of multi-units by 5.0. This result indicates that the multi-unit representation is roughly five times better per unit than the single-unit representation when we do not select high SNR single-units. This remains surprising, as the physical averaging process (averaging multiple single-units in a multi-unit recording) produces a loss of information and may, a priori have been thought to reduce performance. The multi-unit representation is less than five times better than the single-units selected for high SNR, indicating that the multi-unit representation may average single-unit responses without bias for high SNR single-units.

Processing time and energy consumption of computational models

The processing time of DNNs as implemented on current hardware is comparable to that of the macaque and human visual systems. The algorithm of Zeiler & Fergus 2013, which is very similar in implementation to Krizhevsky et al. 2012, utilizes a high-performance GPU and processes batches of 128 images in 8.4 seconds, or 65 ms per image. This processing time is shorter than the presentation times we use during the neural recordings and for our estimate of human performance (both 100 ms). It is also shorter than the integration window we use to measure IT multi-unit responses, which we average between 70 ms

and 170 ms post image onset. Finally, it is also comparable to the latency of response in IT cortex to image presentation (typically 70 ms to 100 ms). However, the 65 ms processing time for the DNN does not include the process of phototransduction (image capture) nor any communications latencies within the computer system (e.g. between system memory and GPU memory). Behavioral response times of macaque to certain discrimination tasks are typically between 230 and 250 ms, and in humans are roughly 50 ms longer (see [4] and [5]). It is likely that current DNN implementations could achieve response times less than those observed in macaque or human.

The energy efficiency of DNNs appears to be far worse than that of macaque or human. Modern high-performance GPUs typically operate between 200 and 350 Watts (W) at load (100 to 125 W at idle). Estimates of the power consumption of the entire human brain are typically around 20 W. Using an estimate that the macaque ventral stream is around 1/40 the size of the human brain (the macaque brain is less than 1/10 the size of the adult human brain and a high estimate of the ventral stream size is 1/4 of the macaque brain), we estimate that macaque ventral stream operates around 0.5 W. Assuming that the macaque brain operates at this power consumption rate during visual behavior, we estimate that the macaque ventral stream is around 400 times or between 2 and 3 orders of magnitude more energy efficient than current DNN implementations (however, see [6] for progress on energy efficient DNN implementations).

References

1. DiCarlo JJ, Zoccolan D, Rust NC (2012) How Does the Brain Solve Visual Object Recognition? Neuron 73: 415–434.

2. Andrews TJ, Coppola DM (1999) Idiosyncratic characteristics of saccadic eye movements when viewing different visual environments. Vision Research 39: 2947 - 2953.

3. Rust NC, DiCarlo JJ (2010) Selectivity and tolerance ("invariance") both increase as visual information propagates from cortical area V4 to IT. Journal of Neuroscience 30: 12978–12995.

4. Thorpe SJ, Fabre-Thorpe M (2001) Perspectives: Neuroscience - Seeking categories in the brain. Science 291: 260–263.

5. Fabre-Thorpe M (2011) The Characteristics and Limits of Rapid Visual Categorization. Frontiers in Psychology 2: 243.

6. Farabet C, Martini B, Corda B, Akselrod P, Culurciello E, et al. (2011) NeuFlow: A runtime reconfigurable dataflow processor for vision. In: IEEE Computer Society Conference on Computer Vision and Pattern Recognition Workshops (CVPRW 2011). pp. 109–116.
